# A Rare Presentation of Penile Calciphylaxis Requiring Partial Penectomy

**DOI:** 10.7759/cureus.28211

**Published:** 2022-08-20

**Authors:** Abdalhai Alshoubi, Asma Matougelwerfelli

**Affiliations:** 1 Clinical Anesthesiology and Critical Care Medicine, St. Joseph’s Medical Center, Stockton, USA; 2 Pediatric Medicine, University of Illinois Peoria, Peoria, USA

**Keywords:** penile calciphylaxis, penectomy, kidney diseases, gangrene, calciphylaxis

## Abstract

Calciphylaxis is a rare disease and carries high morbidity and mortality rates. It’s characterized by microvascular calcification and occlusion, which leads to a life-threatening disease characterized by skin necrosis and ulceration. Calciphylaxis is classified as uremic, which occurs in patients with end-stage renal disease and who are non-uremic. Non-uremic calciphylaxis is an even rarer disease that occurs in patients without end-stage renal disease and has a high mortality rate secondary to sepsis. The most common risk factors are diabetes mellitus, hyperparathyroidism, malignant neoplasm, warfarin-based anticoagulation, alcoholic liver disease, and autoimmune disorders. The management includes wound debridement, pain management, and sepsis control.

We report a case of penile calciphylaxis in a 36-year-old male with a 15-year history of type II diabetes mellitus and chronic kidney disease. He presented with penile ulceration, which rapidly progressed to necrosis. He also had skin necrosis, characteristic of penile calciphylaxis. The patient has perished of multiorgan failure secondary to severe septic shock.

## Introduction

Calciphylaxis, also referred to as calcific uremic arteriolopathy, is a life-threatening disease characterized by skin necrosis and ulceration. Calciphylaxis usually affects the lower extremities. The abdomen and buttocks can also be affected. However, penile calciphylaxis is a rare phenomenon reported in only a few cases in the literature [1,2].

The pathophysiology of penile calciphylaxis includes intimal hyperplasia, inflammation, necrosis, fibrosis, and calcification of the muscular layer of penile small arteries. The management varies according to the clinical presentation. Medical management includes avoiding/treating hypercalcemia and hyperphosphatemia, using non-calcium-containing phosphorous binders, hyperbaric oxygen, and wound care. The systemic antibiotic can be used if there is evidence of infection. Surgical management ranges from simple debridement to penectomy.

We report a case of penile calciphylaxis in a 36-year-old male with a 15-year history of type II diabetes mellitus and chronic kidney disease. He presented with penile ulceration, which rapidly progressed to dry gangrene.

## Case presentation

**Figure 1:**
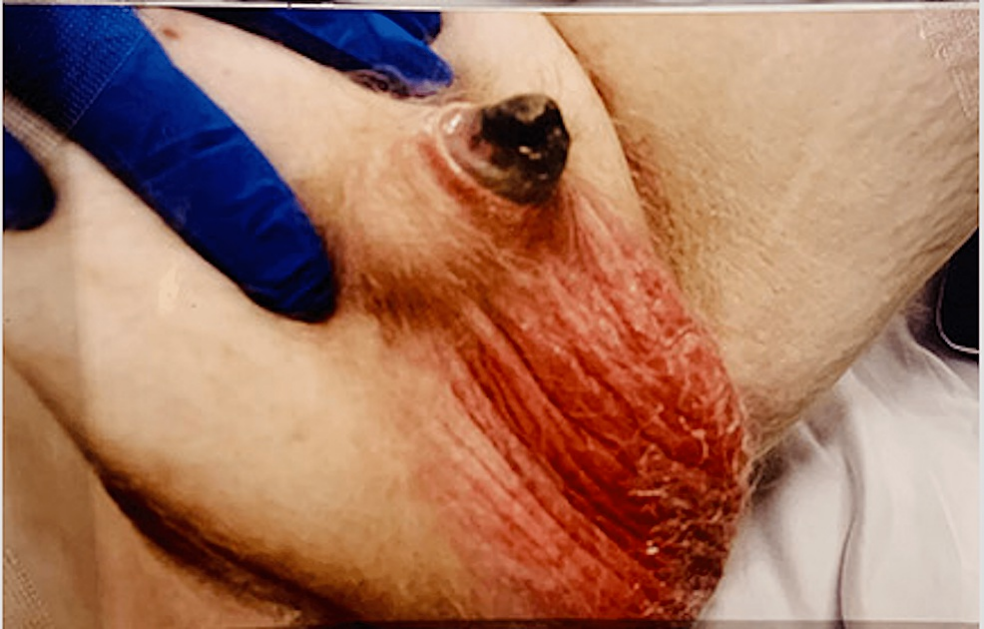
Preoperative appearance of penile dry gangrene and necrosis

A 36-year-old man presented to the emergency department with a three-week history of rapidly developing, painful penile and groin lesions. The patient's height was 181 cm, and his weight was 73 kg with a body mass index of 22.3 kg/m^2^. He had also been having reduced urine output, and a weak stream, which he described as a trickle, his symptoms were associated with mild suprapubic fullness, but no significant abdominal pain, and he denied any nausea, vomiting, fevers, or urethral discharge. The patient had no history of sexually transmitted infections and denied any history of intravenous drug use, alcohol, or tobacco use. He had a history of unspecified arrhythmia for which pacemaker placement was planned, stage IV chronic kidney disease for which he was being evaluated for potential dialysis fistula due to worsening kidney function, type II diabetes mellitus for 15 years, and congestive heart failure. His vital signs showed skin temperature of 38.1 degrees Celsius, respiratory rate of 21 cycles per minute, oxygen saturation of 96% on room air, heart rate of 102 beats per minute, and blood pressure of 110/61 mmHg. The chest X-ray was unremarkable. Physical examination showed a violaceus rash, which progressed to black lesions (Figure 1).

**Figure 2:**
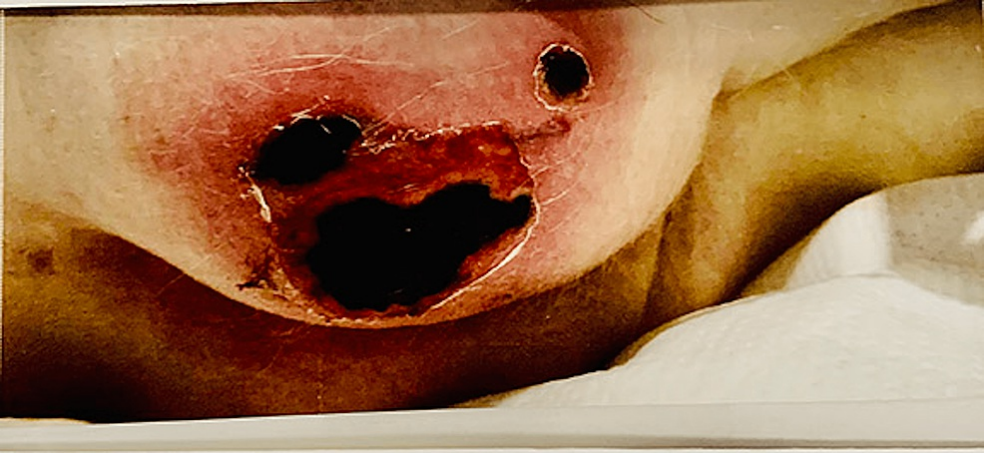
Right buttock skin necrosis, which shows three dry gangrenous ulcers of different sizes

He also had skin necrosis on the right buttock (Figure 2). The rest of his physical examination was unremarkable.

The antinuclear antibody was negative, and complement component 3 and complement component 4 were both within the normal range. Antineutrophil cytoplasmic antibody was positive, which was atypically 1:20. Proteinase 3 and myeloperoxidase were negative. The hepatitis C antibody was positive.

The patient laboratory diagnostics showed evidence of microcytic anemia, leukocytosis with left shift, and elevated neutrophil-lymphocyte ratio. His metabolic panel showed hyponatremia, severe metabolic acidosis, and worsening chronic kidney failure (Table 1).

**Table 1:** Serum laboratory results on the first and last day of admission

Parameter	Reference range and units	First day of admission	Last day of admission
White blood cell count (WBC)	4.0-10.0 10^3/uL	15.9	17.7
Red blood cell count (RBC)	4.3-5.9 10^6/uL	3.6	3.14
Hemoglobin	14.0-18.0 g/dL	8.9	7.5
Hematocrit	39 -49 %	27.8	26.8
Mean corpuscular volume	80.0-99.0 fL	75.8	85.4
Red cell distribution width	11.4-14.6 %	18.5	20.1
Platelet count	150-400 10^3/uL	307	103
Lymphocytes	16.0-45.0%	2	10
Neutrophils relative percent	42.0-75.0%	90	82
Monocytes	2.0-12.0%	6	7
Eosinophils	0.0-5.0%	2	0
Basophils	0.0-2.0%	0.3	1
Sodium	135-145 mmol/L	126	139
Potassium	3.5-5.1 mmol/L	5.6	4.7
Chloride	98-107 mmol/L	93	94
Carbon dioxide	21-32 mmol/L	11	8
Glucose	74-106 mg/dL	237	225
Bloor urea nitrogen (BUN)	7.0-18 mg/dL	135	48
Creatinine	0.70-1.30 mg/dL	10	5.1
Calcium	8.5-10.1 mg/dL	9.8	10.1
Phosphorus	2.3-4.7 mg/dL	10.1	5.7
Aspartate aminotransferase (AST)	15-37 U/L	21	1300
Alanine transaminase (ALT)	16-61 U/L	23	257
Protein, total	6.4-8.2 gm/dL	5.1	4.1
Albumin	3.4-5 gm/dL	2.8	2.6
Alkaline phosphatase	40-150 U/L	172	136
Bilirubin, total	0.3-1.00 mg/dL	0.6	2
International normalized ratio (INR)	1	1	5.4
Prothrombin (PT)	9.4-12.5 seconds	13	65.9
Partial thromboplastin time (PTT)	25.1-36.5 seconds	32	71.2
B-type natriuretic peptide (BNP)	<100 pg/mL	3600	-
Lactic acid	0.5-2 mmol/L	7.5	23.2

**Figure 3:**
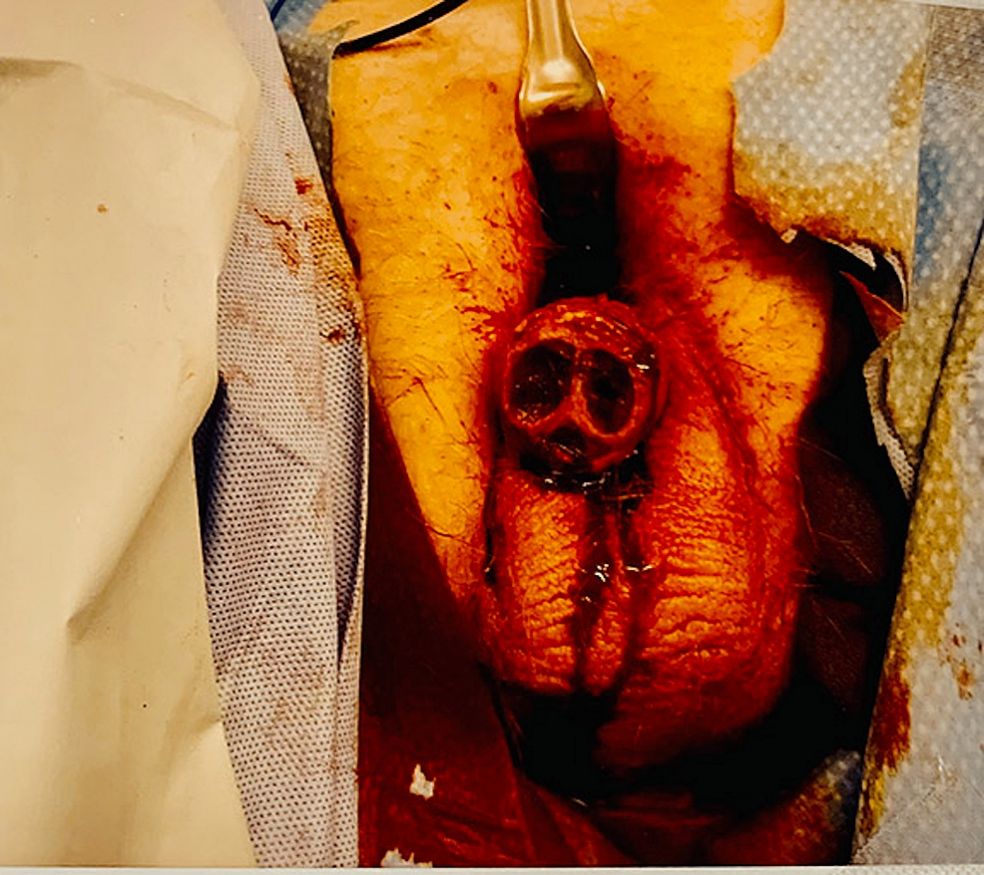
Partial penectomy and removal of necrotic tissues

His medications included baclofen 20 mg oral tablet three times a day (TID), calcium acetate 667 mg tablet TID, carvedilol 25 mg oral tablet two times a day (BID), ferrous sulfate 325 mg oral tablet TID, furosemide 40 mg oral tablet TID, isosorbide mononitrate 60 mg oral tablet BID, lisinopril 5 mg oral tablet once a day, spironolactone 25 mg oral tablet once a day, vitamin D3 2,000 unit once a day, The patient was not on any anticoagulant medications, such as warfarin. One set of blood cultures grew methicillin-resistant Staphylococcus aureus. The differential diagnosis included but was not limited to necrotizing infection, cellulitis/abscess, penile calciphylaxis, and penile cancer. The patient was dialyzed before surgery. Due to the worsening clinical status and progression of necrosis to the distal penis, partial penectomy was performed (Figure 3).

The intraoperative findings showed extensive ischemic damage, with no signs of tissue viability of the spongiosal or cavernosal tissues of the pendulous part of the penis. The remaining tissue was nonviable, so further dissection was done until viable bleeding tissue was seen at the penoscrotal junction. Samples were sent for biopsy, which showed calcification.

A sterile dressing was applied on the remaining stump with a plan for reconstructive surgery later. The patient was admitted to the intensive care unit (ICU) postoperatively. During his ICU stay, the patient developed severe septic shock and required high doses of epinephrine (5 μg/kg/min), norepinephrine (5 μg/kg/min), and vasopressin (0.03 u/min). Four days later, the patient died of multiple organ system failures.

## Discussion

Calciphylaxis is a life-threatening disease characterized by skin necrosis and ulceration secondary to peripheral vascular disease due to the deposition of calcium in the medial layer of small blood vessels. It’s estimated that 1-4% of patients affected have end-stage kidney disease [1,2]. However, penile calciphylaxis has been reported in only a few cases in the literature. It’s a rare disease and the treatment usually is conservative [3], which includes sodium thiosulfate [4], hyperbaric oxygen, internal iliac artery angioplasty, stent placement, vascular bypass, penectomy, and parathyroidectomy [5].

The literature review showed that there was no significant mortality benefit from penectomy and parathyroidectomy [6]. However, since there is no definite curative treatment, physicians may attempt to combine different treatment modalities. Understanding more about this condition may promote more tailored management.

The pathogenesis of calciphylaxis is poorly understood, with multiple theories and several risk factors. The reduced vitamin D level leads to reduced calcium absorption, which in turn triggers a surge in parathyroid hormone, ultimately resulting in the elevation of calcium and phosphate. These metabolic changes are assumed to promote vascular calcifications. Although, it must be noted that calciphylaxis may develop even if parathyroid hormone, phosphorus, and calcium levels are normal [7].

Another theory thought to play a role is the decrease in endogenous calcification inhibitory factors, such as serum fetuin-A, which is down-regulated in dialysis patients, along with an increase in proinflammatory cytokines, such as interleukin 6 and tumor necrosis factor-alpha, which also cause endothelial dysfunction, calcification, and atherosclerosis. Ultimately, this leads to the dysregulation of calcium deposition in cutaneous blood vessels [3]. Warfarin is considered to be a risk factor for calciphylaxis because it inhibits Matrix Gla protein (MGP) activation through the vitamin K-dependent activation process. MGP is considered an essential factor in the prevention of vascular calcification [8].

In a literature review, the average age was 59 years (range 32 to 81). In addition to our case, all patients with calciphylaxis had end-stage kidney disease, the average calcium level was 9.2 mg/dL (the normal range is 8.9-10.3 mg/dL), and the average phosphate level was 8.0 mg/dL (normal range is 2.7-4.5 mg/dL). The overall mortality rate was 56.5%. There was no statistical significance in the correlation between mortality rate and age, diabetes mellitus, calcium level, phosphate level, and calcium-phosphate product level [9]. Histologically, calciphylaxis typically shows calcification of the medial layer of arterioles and small arteries [7]. Endothelial injury and formation of microthrombi lead to luminal narrowing and occlusion, further reducing blood flow and causing tissue ischemia, necrosis, and ulceration [3].

The abdomen, thighs, and buttocks are most commonly involved. It is more common proximally than distally although distal sites, such as the digits and penis, can also be affected. Pain is a hallmark of calciphylaxis, which also leads to considerable morbidity. Penile calciphylaxis is a rare presentation and is associated with a high mortality rate because calciphylaxis involves smaller arteries, arterioles, and capillaries systemically. Unlike other causes of penile gangrene, the benefit of surgical therapy, in this case, is still controversial [6].

Patients with extragenital gangrene have higher rates of mortality. Calciphylaxis can be caused by uremic and non-uremic causes or a combination of both. Determining risk factors and establishing a calciphylaxis risk-scoring system for those with multiple risk factors could help with prevention and early intervention such as stopping medications that may promote calciphylaxis. Reports of patients with decreased protein C or S benefitting from low molecular weight heparin have also been documented [10].

Special attention to patients with liver disease should also be given; these patients can develop non-uremic calciphylaxis, as they may have reduced fetuin-A and MGP [11]. Cryoglobulinemia can also create a microenvironment for calciphylaxis; these patients could benefit from steroids, intravenous immunoglobulin, and plasma exchange [12].

## Conclusions

A high index of suspicion should be present in all patients with end-stage kidney disease, even if they are not on dialysis and who present with characteristic skin and genital lesions. Prevention by careful attention to medications that may promote calciphylaxis such as stopping warfarin, calcium supplements, and iron should be practiced. Control and prevention of tertiary hyperparathyroidism were also shown to be preventive. There is no current definitive treatment or clear guidelines, thus prevention should be prioritized.

## References

[1] El-Taji O, Bondad J, Faruqui S, Bycroft J (2020). Penile calciphylaxis: a conservative approach. Ann R Coll Surg Engl.

[2] Lipinski M, Sahu N (2020). Hyperbaric oxygen therapy improving penile calciphylaxis. Cureus.

[3] Jeong HS, Dominguez AR (2016). Calciphylaxis: controversies in pathogenesis, diagnosis and treatment. Am J Med Sci.

[4] Nigwekar SU, Kroshinsky D, Nazarian RM (2015). Calciphylaxis: risk factors, diagnosis, and treatment. Am J Kidney Dis.

[5] Omer M, Bhat ZY, Fonte N, Imran N, Sondheimer J, Osman-Malik Y (2021). Calcific uremic arteriolopathy: a case series and review from an inner-city tertiary university center in end-stage renal disease patients on renal replacement therapy. Int J Nephrol.

[6] Pollock GR, Zeng J, Gretzer M (2020). Partial penectomy for dry penile gangrene in a patient with penile calciphylaxis. Urology.

[7] Yang TY, Wang TY, Chen M, Sun FJ, Chiu AW, Chen YH (2018). Penile calciphylaxis in a patient with end-stage renal disease: a case report and review of the literature. Open Med (Wars).

[8] Cai MM, Smith ER, Brumby C, McMahon LP, Holt SG (2013). Fetuin-A-containing calciprotein particle levels can be reduced by dialysis, sodium thiosulphate and plasma exchange. Potential therapeutic implications for calciphylaxis?. Nephrology (Carlton).

[9] Kullich W, Machreich K, Hawa G, Eichinger B, Klein G (2003). Calcification marker matrix G1a protein in patients with hyperlipidemia [Article in German]. Wien Med Wochenschr.

[10] Chinnadurai R, Huckle A, Hegarty J, Kalra PA, Sinha S (2021). Calciphylaxis in end-stage kidney disease: outcome data from the United Kingdom Calciphylaxis Study. J Nephrol.

[11] Saifan C, Saad M, El-Charabaty E, El-Sayegh S (2013). Warfarin-induced calciphylaxis: a case report and review of literature. Int J Gen Med.

[12] Siami GA, Siami FS (1999). Intensive tandem cryofiltration apheresis and hemodialysis to treat a patient with severe calciphylaxis, cryoglobulinemia, and end-stage renal disease. ASAIO J.

